# Cancer, psychiatry and homelessness: oncological burden, comorbidity and mortality in a 15-year retrospective cohort in Spain

**DOI:** 10.1192/j.eurpsy.2026.10659

**Published:** 2026-07-31

**Authors:** F. Calvo, S. Font-Mayolas, O. Turró-Garriga

**Affiliations:** 1 Departament de Pedagogia; 2 Departament de Psicologia, Universitat de Girona, Girona, Spain

## Abstract

**Introduction:**

People experiencing homelessness (PEH) present high psychiatric comorbidity and reduced survival. Evidence on cancer among PEH and its intersection with addictions and mental health remains scarce in Spain.

**Objectives:**

1. To estimate cancer burden in PEH.
2. To describe psychiatric comorbidity (substance use disorders [SUD] and other disorders) and its relationship with mortality.
3. To explore differences by sex and origin (Spanish-born vs. foreign-born).

**Methods:**

Secondary analysis of a retrospective cohort (Girona, 2006–2020) of PEH through linkage of clinical records (mental health, addictions, primary care, and specialized services). Diagnoses were coded according to ICD-10/11. Prevalence, causes of death, and previously published regression findings (predictors of age at death) are reported.

**Results:**

Cancer was diagnosed in 4.0% of the cohort (n=118) and accounted for 7.9% of deaths with a known cause. Overall mortality was 13.2%, with a mean age at death of 52.4 years. Psychiatric comorbidity was high: SUD in 31.6% and dual pathology in 11.5%. Multivariable models showed lower age at death associated with SUD (alcohol, opioids, cocaine), COPD, CVD, bipolar disorder, and being Spanish-born; cancer was also linked to earlier death. Women displayed worse health and higher mortality than men; Spanish-born participants died almost nine years earlier than foreign-born.

**Conclusions:**

Among PEH, oncological burden coexists with high psychiatric and addictive comorbidity, driving premature mortality. Clinical practice should integrate opportunistic cancer screening and continuity of care with tobacco and SUD treatment programs, coordinate mental health–oncology–primary care pathways, and tailor interventions by sex and origin (immigrant health advantage). Findings support the need for smoke-free residential services and rapid onco-psychiatric referral pathways for PEH.

**Disclosure of Interest:**

None Declared

